# Efficacy of cognitive behavioural therapy for sleep improvement in patients with persistent delusions and hallucinations (BEST): a prospective, assessor-blind, randomised controlled pilot trial

**DOI:** 10.1016/S2215-0366(15)00314-4

**Published:** 2015-11

**Authors:** Daniel Freeman, Felicity Waite, Helen Startup, Elissa Myers, Rachel Lister, Josephine McInerney, Allison G Harvey, John Geddes, Zenobia Zaiwalla, Ramon Luengo-Fernandez, Russell Foster, Lei Clifton, Ly-Mee Yu

**Affiliations:** a Department of Psychiatry, Warneford Hospital, University of Oxford, Oxford, UK; b Health Economics Research Centre, Department of Public Health, University of Oxford, Oxford, UK; c Sleep and Circadian Neurosciences Institute, Nuffield Department of Clinical Neurosciences, John Radcliffe Hospital, University of Oxford, Oxford, UK; d Centre for Statistics in Medicine, Nuffield Department of Orthopaedics, Rheumatology and Musculoskeletal Sciences, University of Oxford, Oxford, UK; e Nuffield Department of Primary Care Health Sciences, University of Oxford, Oxford, UK; f Sussex Partnership NHS Trust, Worthing, West Sussex; g Department of Psychology, University of California, Berkeley, CA, USA; h Oxford Non-Respiratory Sleep Disorder Service, Oxford University Hospitals NHS Trust, John Radcliffe Hospital, Oxford, UK

## Abstract

**Background:**

Sleep disturbance occurs in most patients with delusions or hallucinations and should be treated as a clinical problem in its own right. However, cognitive behavioural therapy (CBT)—the best evidence-based treatment for insomnia—has not been tested in this patient population. We aimed to pilot procedures for a randomised trial testing CBT for sleep problems in patients with current psychotic experiences, and to provide a preliminary assessment of potential benefit.

**Methods:**

We did this prospective, assessor-blind, randomised controlled pilot trial (Better Sleep Trial [BEST]) at two mental health centres in the UK. Patients (aged 18–65 years) with persistent distressing delusions or hallucinations in the context of insomnia and a schizophrenia spectrum diagnosis were randomly assigned (1:1), via a web-based randomisation system with minimisation to balance for sex, insomnia severity, and psychotic experiences, to receive either eight sessions of CBT plus standard care (medication and contact with the local clinical team) or standard care alone. Research assessors were masked to group allocation. Assessment of outcome was done at weeks 0, 12 (post-treatment), and 24 (follow-up). The primary efficacy outcomes were insomnia assessed by the Insomnia Severity Index (ISI) and delusions and hallucinations assessed by the Psychotic Symptoms Rating Scale (PSYRATS) at week 12. We did analysis by intention to treat, with an aim to provide confidence interval estimation of treatment effects. This study is registered with ISRCTN, number 33695128.

**Findings:**

Between Dec 14, 2012, and May 22, 2013, and Nov 7, 2013, and Aug 26, 2014, we randomly assigned 50 patients to receive CBT plus standard care (n=24) or standard care alone (n=26). The last assessments were completed on Feb 10, 2015. 48 (96%) patients provided follow-up data. 23 (96%) patients offered CBT took up the intervention. Compared with standard care, CBT led to reductions in insomnia in the large effect size range at week 12 (adjusted mean difference 6·1, 95% CI 3·0–9·2, effect size d=1·9). By week 12, nine (41%) of 22 patients receiving CBT and one (4%) of 25 patients receiving standard care alone no longer had insomnia, with ISI scores lower than the cutoff for insomnia. The treatment effect estimation for CBT covered a range from reducing but also increasing delusions (adjusted mean difference 0·3, 95% CI −2·0 to 2·6) and hallucinations (−1·9, −6·5 to 2·7). Three patients, all in the CBT group, had five adverse events, although none were regarded as related to study treatment.

**Interpretation:**

Our findings show that CBT for insomnia might be highly effective for improving sleep in patients with persistent delusions or hallucinations. A larger, suitably powered phase 3 study is now needed to provide a precise estimate of the effects of CBT for sleep problems, both on sleep and psychotic experiences.

**Funding:**

Research for Patient Benefit Programme, National Institute for Health Research.

## Introduction

Sleep problems are pervasive in people with schizophrenia. In a study^1^ of patients with persecutory delusions, 54% had clinical insomnia, 30% had subthreshold insomnia, and only 16% were sleeping well. In outpatients with clinically stable schizophrenia, poor sleep is common^2^, ^3^ and associated with an increased severity of positive symptoms.^3^ Relatives of patients with schizophrenia notice sleep problems more than any other sign preceding relapse,^4^ and a meta-analysis^5^ concluded that “sleep disorders are an intrinsic feature of schizophrenia”. These findings extend into the general population. Findings from two national epidemiological studies^6^, ^7^ have shown that insomnia is strongly associated with paranoia; additionally, sleep problems are associated with psychotic-like experiences in children.^8^ Yet, the treatment (or even routine assessment) of sleep problems in people with schizophrenia has received scant attention. Clinical trials in populations without a diagnosis of schizophrenia have shown that cognitive behavioural therapy (CBT) is highly effective for treatment of insomnia,^9^, ^10^, ^11^, ^12^ and CBT is considered by many as the recommended treatment for this disorder.^13^ However, this therapy is yet to be tested in a randomised controlled trial in patients with schizophrenia.

**Research in context**

**Evidence before this study**

We searched PubMed and the ISRCTN trial registry up to April 10, 2015, with the terms “insomnia”, “therapy”, and “schizophrenia”, without date restrictions, for English-language publications of randomised controlled trials investigating the treatment of insomnia with patients with schizophrenia. We did not find any randomised trials of treatment of insomnia in schizophrenia with a psychological therapy. Apart from our own case report series, we did not find any descriptions of cognitive behavioural therapy (CBT) for insomnia with patients with schizophrenia. Three further searches with the terms “insomnia”, “randomized”, “schizophrenia”, and “hypnotics/benzodiazepine/melatonin”, and reading of review papers, identified only three small randomised controlled trials testing the short-term effects of melatonin and eszopiclone. We found no randomised controlled trials assessing treatment of insomnia in patients selected for having current delusions and hallucinations.

**Added value of this study**

Our trial is the first randomised controlled trial to treat insomnia in patients with current psychotic experiences, use a psychological treatment for insomnia with patients with schizophrenia, and examine effects of an insomnia treatment for patients with schizophrenia for up to 6 months. Our findings show that CBT for insomnia is likely to be beneficial for reducing insomnia in patients with schizophrenia.

**Implications of all the available evidence**

Trial data are insufficient for the treatment of sleep problems in patients with schizophrenia. The present study, in combination with studies of CBT for insomnia in other disorders, suggests that CBT could be offered as psychological treatment for patients with schizophrenia. However a larger, suitably powered phase 3 study is indicated.

Treatment of sleep problems in patients with psychosis might have another important benefit: reductions in delusions and hallucinations. Sleep disturbance is increasingly recognised as a potential contributory factor to the occurrence of a wide range of mental health problems.^14^, ^15^ Sleep disturbance might also have a role in the occurrence of psychotic experiences such as delusions and hallucinations. Findings from longitudinal studies have shown that insomnia predicts new inceptions of paranoia^16^ and its persistence.^17^ Insomnia increases negative affect, anomalous perceptions, and reasoning errors, which are all factors implicated in the development of persecutory ideation. Studies have also linked insomnia and hallucinatory experiences.^18^, ^19^ In adolescents at an ultra-high risk of psychosis, sleep difficulties are a predictor of positive psychotic experiences.^20^ Importantly, research in twins has shown overlap in the genetic and environmental causes of insomnia and psychotic experiences.^18^ If a causal link does exist, the clinical implication is that treatment of insomnia in patients with schizophrenia could lessen psychotic experiences, which would provide a new treatment route for these patients.

In a case series,^21^ we used CBT for insomnia in 15 patients with persistent persecutory delusions in the context of non-affective psychosis. After the brief CBT intervention, we recorded large reductions in levels of insomnia and paranoia. We also recorded significant reductions in levels of hallucinations, anxiety, and depression. A methodologically rigorous assessment is now needed. We planned the Better Sleep Trial (BEST) as a randomised controlled pilot trial assessing treatment of insomnia in patients with persistent delusions or hallucinations. We followed the recommendation of Lee and colleagues^22^ that “pilot studies are more about learning than confirming: they are not designed to formally assess evidence of benefit”. As such, we aimed to establish recruitment and follow-up rates, indicate levels of compliance with the treatment, examine the use of sleep assessments in this population, and provide an estimation of the potential efficacy of the intervention.

The study is part of our process of improving treatments for patients with delusions and hallucinations, translating advances in understanding into intervention. One putative causal factor at a time is targeted and the effect on the psychotic experiences examined^23^—a so-called interventionist-causal model approach.^24^ Our intention was to use clinical techniques focused on sleep and then examine the subsequent effects. In this mechanistic approach, the central need is to establish change in the putative causal factor (ie, sleep) in one group compared with a group in which sleep patterns remain relatively stable. Therefore, the appropriate design was to compare the targeting of sleep with standard care. Identification of the active ingredients of intervention was not the question that the trial was designed to address.

We had two primary outcome hypotheses: that CBT for insomnia added to standard care would improve sleep in patients with psychosis compared with standard care alone, and that CBT for insomnia added to standard care would reduce delusions and hallucinations compared with standard care alone. Our secondary hypotheses were that improvements in sleep and psychotic symptoms would be maintained over at least 3 months, that improvement in sleep would be associated with improvements in psychotic symptoms, and that CBT for insomnia would lead to improvements in other outcomes, such as patient wellbeing and feelings of fatigue.

## Methods

### Study design and patients

We did this prospective, assessor-blind, randomised controlled pilot study at two centres in the UK: Oxford Health National Health Service (NHS) Foundation Trust—a large mental health service covering a population of roughly 1·2 million people—and, in the final 4 months of recruitment, at an additional site at Northamptonshire Healthcare NHS Foundation Trust. We sought to include patients who had persistent, distressing delusions or hallucinations in the context of non-affective psychosis and insomnia. Inclusion criteria were current delusion or hallucination; a score of at least 2 on the distress items of the Psychotic Symptoms Rating Scale (PSYRATS)^25^ for either a delusion or hallucination; delusion or hallucination that had persisted for at least 3 months; a clinical diagnosis of schizophrenia, schizoaffective disorder, or delusional disorder (ie, a diagnosis of non-affective psychosis); sleep difficulties lasting 1 month or longer and a score of 15 or more on the Insomnia Severity Index^26^ (ISI; ie, clinical insomnia); an age of 18–65 years; and a medication dosage that had been stable for at least the past month. Exclusion criteria were a primary diagnosis of sleep apnoea, alcohol or substance dependency, organic syndrome or learning disability, a command of spoken English inadequate for engagement in therapy, and current engagement in individual CBT. Patient enrolment was done by one full-time graduate psychologist (RL).

The trial received ethics approval from the NHS Research Ethics Committee South Central–Oxford C (reference 12/SC/0138) and the protocol has been published elsewhere.^27^ All patients provided written informed consent.

### Randomisation and masking

We randomly assigned patients (1:1), via a web-based randomisation system, to receive either CBT plus standard care or standard care alone. The randomisation system was designed to balance three variables with a non-deterministic minimisation algorithm: sex (male *vs* female), severity of sleep problem (moderate [ISI score 15–21] *vs* high [22–28]), and psychotic experiences (hallucination only *vs* delusion only *vs* hallucinations and delusions).

Research assessors (RL for most of the trial, with the last follow-up assessments completed by a replacement graduate psychologist [JM]) were masked to group allocation; the trial therapists informed patients of the randomisation outcome to maintain allocation concealment. Precautionary strategies to prevent unmasking included the therapist and assessor considering room use and booking arrangements; patients being reminded by the assessor not to talk about treatment allocation; and, after the initial assessment, the assessor not looking at the patient's clinical notes. In the case of an allocation being revealed, we remasked by using another assessor (which happened six times in total, three times at each follow-up point). All assessments were therefore done blind to allocation.

### Procedures

The CBT intervention was provided one to one by clinical psychologists (EM and FW), either in NHS clinics or at the patient's home. DF and HS did weekly clinical supervision. The aim was to provide the insomnia intervention in about eight sessions over 12 weeks, with four sessions defined as a minimum therapeutic dose, with flexibility in length and number of sessions as appropriate in this clinical group. We also included telephone calls and texts between sessions to maintain treatment momentum. The main techniques standard for CBT sleep interventions were taken from four main sources.^28^, ^29^, ^30^, ^31^ The intervention was written in a manual to guide the work, which was shared with the patient. Initially the sessions focused on psycho-education about sleep difficulties, assessment of the triggering and maintenance of sleep difficulties, and goal setting. We used a checklist of factors likely to cause sleep difficulties, which was generated by the team. On the basis of the assessment, the active therapeutic techniques used could have included stimulus control therapy (eg, setting of appropriate and regular sleep times, ensuring the bed or bedroom were used only for sleeping, not staying in bed if unable to sleep for longer than 20–30 min, reducing sleep in the daytime), establishment of appropriate daytime activity and circadian rhythms (eg, obtaining natural light in the morning, regular mealtimes, gradually shifting sleep and wake times for sleep-phase problems), sleep hygiene, relaxation, and cognitive techniques addressing unhelpful beliefs and attitudes about sleep. The intervention was deliberately simplified, with the principal therapeutic techniques being stimulus control (ie, learning to associate bed with sleep) and improvement of daytime activity levels. A detailed description of our approach to treatment of sleep problems in this group is available elsewhere,^32^ and includes noting of adaptations needed for the particular problems of delusions and hallucinations interfering with sleep, attempts to sleep being overused by patients as an escape from voices, extensive disruption of circadian rhythms, insufficient daytime activity, and fear of the bed based on past adverse experiences. Sessions were taped with patient agreement. To assess treatment fidelity, six tapes, chosen at random, were rated on the Cognitive Therapy Scale–Revised^33^ by an independent clinical psychologist experienced in CBT for psychosis. All tapes were rated as providing at least satisfactory cognitive therapy (ie, an average score of at least three on scale items). Standard care was delivered according to national and local service protocols and guidelines and mainly consisted of antipsychotic medication and contact with the local clinical team. We recorded medication and hospital admissions from clinical notes and other service provision using a modified version of the Client Service Receipt Inventory.^34^ In the baseline assessment, the trial patients were also assessed for insomnia using the Duke Structured Interview Schedule for Sleep Disorder Diagnoses.^35^

### Outcomes

As a pilot study, our main outcomes concerned the number of participants who were recruited, complied with treatment, and followed up. The prespecified primary outcome measures were levels of insomnia, assessed with the ISI,^26^ and levels of delusions and hallucinations, assessed with the PSYRATS.^25^ Higher scores on these measures indicate greater severity.

Secondary outcome measures assessed sleep using different methods: a second self-report questionnaire measure of sleep (Pittsburgh Sleep Quality Index [PSQI]),^36^ a sleep diary, and actigraphy data obtained by participants wearing an actiwatch (CamNtech MotionWatch 8, CamNtech, Cambridge, UK) for at least 7 days. Additionally, we assessed secondary outcome measures for psychiatric symptoms: a self-report measure of paranoid thinking (Paranoid Thoughts Scale);^37^ a standard psychiatric interviewer-rated assessment (the Positive and Negative Syndromes Scale; PANSS);^38^ and an adapted patient reported outcome measure (CHoice of Outcome In Cbt for psychosEs),^39^ which assessed, for example, self-confidence, peace of mind, and a sense of being in control. Other secondary outcomes were fatigue (Multidimensional Fatigue Inventory),^40^ quality of life (Euroqol 5 Dimensions 5 Levels; responses were converted into a single summary measure using UK population tariffs),^41^ and psychological wellbeing (Warwick-Edinburgh Mental Well-being Scale).^42^ We included several measures, such as the Beck Depression Inventory (BDI),^43^ for the purposes of potential mediation analysis. The outcome measures were completed at weeks 0 (baseline), 12 (post-intervention), and 24 (follow-up).

During the trial, we recorded any adverse event that came to our attention. Medical notes were also checked at the end of the trial for the following events prespecified as adverse: all deaths, suicide attempts, serious violent incidents, admissions to secure units, formal complaints about therapy.

### Statistical analysis

The trial statisticians (LC and L-MY) prepared a fully detailed statistical analysis plan and the chief investigator (DF) approved the plan before any analysis. No formal sample size calculation was done for this pilot study. However, the target sample size was based on recruitment for 15 months at an estimated rate of four patients per month with one research worker (ie, 60 patients), which was considered adequate to obtain reasonably reliable sample size estimates.^44^ Outcomes were assessed separately for assessment points at weeks 12 and 24. We used ANCOVA to obtain estimates and 95% CIs of continuous outcomes, with adjustment for baseline variables. The analysis plan did not include reporting of p values, in accordance with recommendations that “The analysis of a pilot study should be mainly descriptive or should focus on confidence interval estimation.”^45^ As a sensitivity analysis, we controlled for initial overall symptom severity as assessed by the PANSS and use of antipsychotic medication. In a post-hoc analysis for the main outcomes, we constructed a mixed model to incorporate the repeated measures at the two assessments (weeks 12 and 24) for each patient. We calculated effect sizes with Cohen's d by taking the estimated coefficient of treatment allocation from the ANCOVA divided by the pooled baseline SD. An effect size of 0·3 is considered a small effect, 0·5 a medium effect, and 0·8 or higher a large effect. We used correlation coefficients to examine potential associations between changes in sleep and changes in psychotic experiences (changes in scores for each measure were calculated as week 12 score minus baseline score). All main analyses were done at the end of the last follow-up assessments (ie, there were no interim analyses) and were based on the intention-to-treat population. Analyses were done with SAS (version 9.3)^46^ and were repeated by an independent statistician. This study is registered with ISRCTN, number 33695128.

### Role of the funding source

The funder of the study had no role in study design, data collection, data analysis, data interpretation, or writing of the report. DF, L-MY, and LC had full access to all the data in the study and had final responsibility for the decision to submit for publication.

## Results

The figure shows the trial profile. Between Dec 14, 2012, and May 22, 2013, and Nov 7, 2013, and Aug 26, 2014, we randomly assigned 50 patients to receive CBT plus standard care (n=24) or standard care alone (n=26), with the break in enrolment due to employment of a new trial therapist. 47 (94%) patients were from Oxford Health and three (6%) were from Northamptonshire Healthcare. The last assessments were completed on Feb 10, 2015. All 50 patients completed the baseline assessment. 48 (96%) patients provided follow-up data for the primary efficacy measures (figure). In the CBT group, the mean number of sessions received was 7·3 (SD 1·9). On the basis of at least four CBT sessions constituting a minimum therapeutic dose, 23 (96%) of 24 patients had a dose of the intervention. The actual number of treatment sessions attended in the 12 week period was three (n=1), four (n=1), five (n=1), six (n=4), seven (n=5), eight (n=7), nine (n=2), ten (n=2), or 11 (n=1) sessions. The appendix shows descriptive comments about the intervention received from five of the first seven patients who had CBT.

Baseline and demographic characteristics were similar between groups (table 1). In line with other studies of persistent psychotic experiences, both groups included slightly more men, the average age was about 40 years, most participants were unemployed, and the main diagnosis was schizophrenia (table 1). There were high levels of depression in both groups (table 1), although BDI scores were not significantly correlated with level of insomnia at baseline (*r*=0·07, p=0·623). All but one patient (in the standard care alone group) met the Duke Structured Interview Schedule for Sleep Disorder Diagnoses criteria for insomnia disorder. Provision of standard care was similar between groups and was fairly stable during the trial (table 2). All but four participants (n=3 given CBT, n=1 given standard care alone) were taking antipsychotic medication (table 2). Most participants were also prescribed a hypnotic, anxiolytic, or antidepressant medication (19 [79%] in the CBT group, 21 [81%] in the standard care alone group; appendix). All participants were outpatients.

Compared with standard care alone, CBT had a treatment benefit on insomnia in the large effect size range at 12 weeks (table 3). Benefits were still apparent at 24 weeks' follow-up (table 2). At baseline, no patients' ISI scores were lower than the cutoff indicating normal sleep (a score of 0–7). By week 12, nine (41%) of 22 patients in the CBT group and one (4%) of 25 patients in the standard care alone group scored less than this cutoff. The wide confidence intervals for the effects of CBT on delusions and hallucinations cover a range from decreasing to increasing psychotic experiences (table 3). Changes in the other sleep assessments were relatively consistent with those for the primary outcome measure of insomnia (table 3). We recorded moderate to large effect sizes in sleep quality as assessed by the PSQI (table 3). Data collection was less complete for the sleep diaries and actigraphy than for the other secondary outcome measures, and the ensuing effect sizes were small to moderate (table 3). Compared with standard care alone, patients reported reduced fatigue at week 12, and improved quality of life and psychological wellbeing at week 24, with small to medium effect sizes overall for both these categories (table 3). The confidence intervals for paranoia and overall psychiatric symptomatology again span CBT potentially reducing or increasing these problems (table 3). Correlations between changes in insomnia and changes in hallucinations and paranoia (although not PSYRATS delusions) were mainly small and positive, but the confidence intervals are wide and include negative correlations (appendix).

All the notes of the trial patients were checked. Four patients were admitted to hospital during the trial (n=2 in each group; appendix). There were no deaths or complaints about therapy. Three patients, all in the CBT group, had a total of five adverse events: two suicide attempts, two serious violent incidents, and one admission to a secure unit (following one of the violent incidents). No adverse events were considered to be related to study treatment.

The appendix shows results of the sensitivity analysis. Results of the post-hoc analysis using a mixed model for repeated measures analysis were similar to those obtained using ANCOVA (appendix).

## Discussion

This is the first randomised controlled trial testing the effects of CBT for insomnia in patients with current psychotic experiences, which comprised very long-standing insomnia, delusions, and hallucinations. Overall our study shows the feasibility of testing the psychological treatment adapted for this population: it was possible to recruit patients to the trial, to randomise them, to keep assessors masked to allocation, to retain patients in the trial, to assess a wide range of measures, and to implement the treatment. Indeed, there was a very low dropout rate for the trial assessments and a very high uptake of CBT. Our findings also show that CBT for insomnia, a relatively brief intervention, is likely to have substantial benefits in improving sleep for patients with current delusions and hallucinations. Benefits in sleep were sustained up to the final follow-up assessment.

Findings from a qualitative study^47^ of a subsample of the trial participants are consistent with those of the present quantitative analysis. Our results compare well with the only randomised controlled trial that we are aware of that targeted insomnia in patients with schizophrenia with one of the commonly prescribed sedative hypnotics (so-called Z-drugs). In a randomised double-blind trial^48^ done over 8 weeks in 39 clinically stable outpatients with schizophrenia who had insomnia but were not recruited for current psychotic experiences, eszoplicone was compared with placebo. The medication led to a decrease in ISI scores of 3·78 (95% CI 0·2–7·5) versus placebo. The reduction in ISI score in the present study is very similar to that reported in a meta-analysis of CBT for comorbid insomnia for patients with physical or psychiatric disorders (ISI mean change 6·4 [SE 1·27]).^11^ Our trial did not prove informative about the potential effect of the sleep treatment on the psychotic experiences. The wide confidence intervals include the possibility that the treatment can either reduce or increase delusions and hallucinations. A larger trial will provide a better estimate of the effects on psychotic experiences.

The clear caution when discussing potential clinical effects is that this study was a pilot study. Our trial is insufficiently powered to detect anything but the largest effect sizes. There was no formal power calculation because we did not primarily intend to detect treatment effects. We did not achieve the target sample size of 60 patients, but this information will form part of the recruitment strategy plan for the future phase 3 trial. Our main purpose was to assess the feasibility of the assessment and to refine procedures. A key observation was the wide variety of sleep disturbance within the patient group, including patients going to bed early in the evening and simply lying awake for hours, patients not having a bed, patients having an association with their bed as a place of trauma, patients awake and pacing all night, patients' sleep being disturbed through the use of hypnotics, patients being drowsy and oversleeping at various times of the day, and patients only going to bed in the early hours (delayed-phase sleep), whereas others had obviously irregular sleep–wake patterns. Our treatment manual will become more specific to each of these types of presentation and it would be of interest to establish whether they are associated with different outcomes. Such presentations are consistent with evidence of varied circadian rhythm dysfunction in patients with schizophrenia^49^ and, indeed, the complexity of sleep disturbance beyond insomnia in other disorders such as bipolar disorder.^50^ Notably, our trial did not include polysomnography. Obvious practical difficulties exist in implementation of such a measurement, and subjective reports of problems must be the key clinical priority. Instead of polysomnography we used actigraphy to assess sleep–wake timing and fragmentation. This form of assessment (wearing a watch-like device) was not agreed to by all participants. Furthermore, we have concerns that the movement recordings are not able to differentiate between actual sleep and drowsy but awake inactivity, which is common in this patient group. Such recordings might be better used to assess changes in overall activity levels, which is an important outcome in itself. Sleep improvement might also lead to physical health benefits.^51^

The key efficacy question addressed in the design of the present study was whether CBT can improve sleep in patients with current psychotic experiences and insomnia. We assessed this question in the context of our translational research that aims to improve treatments for people with psychosis.^23^ The causal framework that we use leads to the initial aim of showing change in the targeted mechanism (eg, sleep disruption), hence the comparison of a sleep treatment (whereby the expectation is that sleep will improve) with continued standard care (whereby sleep patterns are likely to remain stable). The chosen design also addresses the important clinical question of the potential overall benefit provided when the intervention is added to standard care. From some perspectives, this question is key: “The primary question facing clinicians and policy makers is whether adding a particular form of treatment will significantly improve symptoms or functioning compared to the often-constrained services as usual.”^52^ However, this question would only establish whether the extra treatment has benefits, not which elements of the package are actually necessary or whether there are better forms of treatment. These latter questions require different choices of control group in order to answer them. Nonetheless, our clinical impression is that, for example, the extra contact time alone provided by the CBT is an insufficient explanation for such large improvements in sleep in a patient group that typically has long-standing and complex problems. Indeed, CBT has already been shown to have benefit compared with attention-control conditions in the treatment of primary insomnia.^53^

Sleep difficulties are a problem that patients with psychosis find distressing and very much wish to receive effective treatment for from mental health services. A large, multicentre trial is now needed that can establish the effects of CBT for insomnia, delivered by front-line mental health staff, both on sleep and psychotic experiences.

## Figures and Tables

**Figure:**
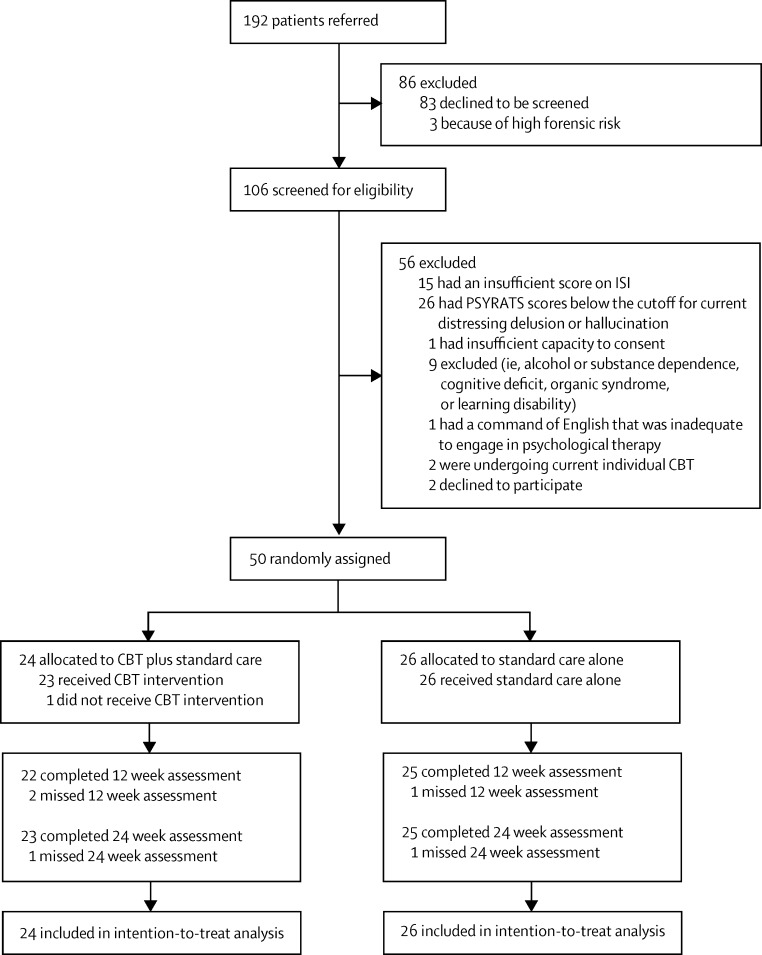
Trial profile ISI=Insomnia Severity Index. PSYRATS=Psychotic Symptoms Rating Scale. CBT=cognitive behavioural therapy.

**Table 1:** Baseline demographic and clinical characteristics

		**CBT group (n=24)**	**Standard care alone group (n=26)**
Age (years)		39·6 (11·6)	42·2 (13·5)
Sex			
	Male	16 (67%)	18 (69%)
	Female	8 (33%)	8 (31%)
Ethnic origin			
	White	22 (92%)	25 (96%)
	Black	1 (4%)	1 (4%)
	Chinese	1 (4%)	0
Employment status			
	Unemployed	21 (88%)	23 (88%)
	Part-time employed	1 (4%)	0
	Full-time employed	0	1 (4%)
	Volunteer	1 (4%)	1 (4%)
	Retired	1 (4%)	0
	Student	0	1 (4%)
Clinical diagnosis			
	Schizophrenia	16 (67%)	17 (65%)
	Schizoaffective disorder	5 (21%)	5 (19%)
	Delusional disorder	0	0
	Psychosis not otherwise specified	3 (13%)	4 (15%)
Severity of insomnia (ISI)			
	Moderate (15–21)	19 (79%)	19 (73%)
	High (22–28)	5 (21%)	7 (27%)
Presence of psychotic experiences			
	Both delusions and hallucinations	18 (75%)	19 (73%)
	Delusion only	4 (17%)	3 (12%)
	Hallucination only	2 (8%)	4 (15%)
Depression (BDI)			
	None (0–13)	3 (13%)	3 (12%)
	Mild (14–19)	5 (21%)	3 (12%)
	Moderate (20–28)	2 (8%)	6 (23%)
	Severe (29–63)	14 (58%)	14 (54%)

Data are mean (SD) or n (%), unless otherwise indicated. CBT=cognitive behavioural therapy. ISI=Insomnia Severity Index. BDI=Beck Depression Inventory.

**Table 2:** Provision of standard care

	**CBT group**		**Standard care alone group**	
	n	Mean (SD)	n	Mean (SD)
**Antipsychotic medication (chlorpromazine equivalent dose, mg/day)**				
Baseline	24	363·7 (266·5)	26	495·8 (358·1)
12 weeks	23	359·8 (289·3)	25	530·7 (366·0)
24 weeks	23	372·5 (302·9)	25	531·3 (373·2)
**6 months before the trial (n)**				
Psychiatric hospital admission	24	0·1 (0·3)	26	0·1 (0·3)
Psychiatrist meetings	23	1·2 (1·4)	26	2·5 (4·9)
Meetings with community psychiatric nurse	23	11·7 (13·6)	26	15·4 (16·2)
Visits to day-care centre	23	6·0 (14·8)	26	2·0 (6·8)
Meetings with general practitioner	22	2·8 (3·6)	25	3·0 (5·3)
**6 months during the trial (n)**				
Psychiatric hospital admission	24	0·1 (0·3)	26	0·1 (0·3)
Psychiatrist meetings	19	1·1 (0·9)	24	2·3 (3·9)
Meetings with community psychiatric nurse	19	7·6 (8·2)	24	13·5 (13·9)
Visits to day-care centre	19	6·1 (18·6)	24	7·9 (24·9)
Meetings with general practitioner	18	3·5 (3·9)	24	3·2 (3·2)

**Table 3:** Scores for the primary and secondary outcome measures

		**CBT group**		**Standard care alone group**		**Adjusted mean difference (95% CI)**	**Effect size (d)**
		n	Mean (SD)	n	Mean (SD)		
**Primary outcome measure**							
Insomnia (ISI)							
	0 weeks	24	18·6 (3·2)	26	18·8 (3·3)	..	..
	12 weeks	22	9·3 (5·5)	25	15·4 (5·4)	6·1 (3·0 to 9·22)	1·9
	24 weeks	23	11·0 (5·6)	25	15·0 (5·7)	3·9 (0·9 to 6·8)	1·2
Delusions (PSYRATS)							
	0 weeks	24	16·1 (3·2)	26	15·3 (4·9)	..	..
	12 weeks	22	13·9 (4·8)	25	13·8 (4·1)	0·3 (−2·0 to 2·6)	0·1
	24 weeks	23	14·0 (4·7)	25	12·7 (5·7)	−0·8 (−3·6 to 2·1)	−0·2
Hallucinations (PSYRATS)							
	0 weeks	24	25·1 (12·1)	26	26·7 (9·2)	..	..
	12 weeks	22	27·5 (9·2)	25	25·9 (8·1)	−1·9 (−6·5 to 2·7)	−0·2
	24 weeks	23	24·6 (11·6)	25	22·0 (10·2)	−3·4 (−9·2 to 2·3)	−0·3
**Sleep secondary outcome measures**							
Sleep quality (PSQI)							
	0 weeks	23	11·9 (3·2)	23	11·6 (2·9)	..	..
	12 weeks	21	8·7 (3·8)	23	10·5 (4·8)	1·9 (−0·4 to 4·1)	0·6
	24 weeks	23	6·9 (4·4)	23	9·6 (4·8)	2·8 (0·2 to 5·4)	0·9
Time to sleep onset (min)*							
	0 weeks	21	61·5 (51·4)	25	61·1 (32·7)	..	..
	12 weeks	22	34·6 (40·3)	19	46·6 (37·0)	18·3 (−1·5 to 38·2)	0·4
	24 weeks	20	33·3 (29·6)	21	57·9 (41·7)	26·4 (2·5 to 50·3)	0·6
Total sleep time (min)*							
	0 weeks	18	393·7 (127·8)	21	403·4 (146·3)	..	..
	12 weeks	17	456·4 (107·7)	18	437·5 (118·1)	33·0 (−17·9 to 83·8)	0·2
	24 weeks	18	465·9 (117·1)	20	412·4 (128·2)	46·6 (−15·1 to 108·3)	0·3
Waking in night (min)*							
	0 weeks	19	43·5 (40·2)	22	59·1 (63·3)	..	..
	12 weeks	18	19·9 (22·2)	18	47·6 (52·1)	16·9 (−4·7 to 38·5)	0·3
	24 weeks	18	32·7 (35·7)	20	42·7 (46·6)	14·6 (−11·1 to 40·3)	0·3
Total sleep time (min)†							
	0 weeks	20	396·3 (141·7)	23	470·0 (128·7)	..	..
	12 weeks	18	406·2 (100·0)	19	471·0 (124·4)	−15·5 (−78·9 to 47·9)	−0·1
	24 weeks	18	445·1 (86·7)	21	449·8 (141·1)	33·1 (−27·0 to 93·3)	0·2
**Psychiatric secondary measures**							
Paranoia (GPTS)							
	0 weeks	24	90·8 (28·7)	26	90·5 (29·8)	..	..
	12 weeks	22	89·6 (36·8)	24	96·2 (37·3)	6·7 (−10·2 to 23·5)	0·2
	24 weeks	20	78·3 (34·8)	25	88·1 (35·0)	9·8 (−7·5 to 27·2)	0·3
Total symptoms (PANSS)							
	0 weeks	24	83·6 (16·2)	26	79·7 (14·1)	..	..
	12 weeks	22	77·5 (12·1)	24	79·3 (14·6)	3·4 (−2·3 to 9·0)	0·2
	24 weeks	21	74·8 (14·7)	24	75·8 (11·8)	3·3 (−2·9 to 9·5)	0·2
Fatigue (MFI)							
	0 weeks	23	43·8 (16·4)	26	47·6 (15·3)	..	..
	12 weeks	22	29·1 (19·0)	24	45·4 (19·6)	10·5 (2·1 to 18·9)	0·7
	24 weeks	21	25·9 (21·4)	25	38·4 (18·1)	9·0 (−1·1 to 19·1)	0·6
Patient outcomes (CHOICE)							
	0 weeks	23	52·2 (21·1)	26	55·0 (14·4)	..	..
	12 weeks	22	58·0 (22·7)	24	49·9 (18·3)	8·5 (−1·4 to 18·5)	0·5
	24 weeks	21	60·0 (22·8)	23	57·5 (21·8)	3·8 (−7·3 to 14·9)	0·2
Quality of life (EQ-5D-5L)							
	0 weeks	24	0·55 (0·23)	26	0·60 (0·22)	..	..
	12 weeks	22	0·63 (0·25)	24	0·55 (0·22)	0·11 (0·02 to 0·23)	0·5
	24 weeks	22	0·63 (0·27)	25	0·58 (0·20)	0·08 (−0·05 to 0·21)	0·4
Wellbeing (WEMWBS)							
	0 weeks	24	35·3 (9·3)	26	37·0 (7·8)	..	..
	12 weeks	22	36·1 (10·7)	24	34·0 (8·9)	2·6 (−2·1 to 7·4)	0·3
	24 weeks	21	39·4 (9·9)	25	34·7 (7·9)	4·8 (−0·5 to 9·3)	0·6

CBT=cognitive behavioural therapy. ISI=Insomnia Severity Index. PSYRATS=Psychotic Symptoms Rating Scale. PSQI=Pittsburgh Sleep Quality Index. GPTS=Green et al Paranoid Thought Scales. PANSS=Positive and Negative Syndromes Scale. MFI=Multidimensional Fatigue Inventory. CHOICE=CHoice of Outcome In Cbt for psychoses. EQ–5D=Euroqol 5 Dimensions 5 Levels. WEMWBS=Warwick-Edinburgh Mental Well-being Scale.

* Sleep diary.

† Actigraphy data.
