# Supplementary material for: Efficacy of cognitive behavioural therapy for sleep improvement in patients with persistent delusions and hallucinations (BEST): a prospective, assessor-blind, randomised controlled pilot trial
Source: Lancet Psychiatry. 2015 Nov;2(11):975–83. doi: 10.1016/S2215-0366(15)00314-4 (PMC4641164; doi:10.1016/S2215-0366(15)00314-4)
Supplement: Supplementary appendix [file mmc1.pdf]

THE LANCET Psychiatry

Supplementary appendix

This appendix formed part of the original submission and has been peer reviewed. We post it as supplied by the authors.

Supplement to: Freeman D, Waite F, Startup H, et al. Efficacy of cognitive behavioural therapy for sleep improvement in patients with persistent delusions and hallucinations (BEST): a prospective, assessor-blind, randomised controlled pilot trial. *Lancet Psychiatry* 2015; published online Sept 10. http://dx.doi.org/10.1016/S2215-0366(15)00314-4.

Descriptive comments about the CBT intervention

Patient 1

"I wasn't sure to begin with–I am not very good with new people and that. I was really unsure to start with, nothing else had worked and I had tried loads of stuff, but the more I tried it the more impressed I was with it. It really made a difference…I am asleep within half an hour of going to bed. I wake up occasionally but not every night and then I will get up at 6–7 in the morning feeling quite refreshed and not tired at all."

Patient 2

"Yes I sleep a bit longer now, I sleep about 4 or 5 hours now and it doesn't take me so long to get to sleep. I wake up feeling a lot better in the morning now than I used to, although I didn't used to feel bad in the mornings I now feel a lot more awake and a lot fresher. I don't wake up as much in the middle of the night now either…I have now started getting a lot more work coming in because I have now got the motivation to go out and start showing people what I can do. So I am getting a lot more work coming in. I have signed up to go back to college to go back into education and learn again, because before the treatment I wasn't sure how I would be feeling and whether the stress would bother me again and whether the sleep would help or not. So it has given me a lot more motivation and confidence within myself."

Patient 3

"Yes I get tired easily, but I feel much better now I have got a better routine and night sleep. I stay asleep a lot better now as well. If I have nightmares I just think 'oh it is just my brain processing it' and I don't worry about them anymore, before I used to worry about them…I think it is better because I feel that I can do things, I am up earlier and I just feel better. I can cope a bit better because I am not asleep all day and I can do things. I feel a bit more normal.

Patient 4

"Yes she told me not to have an afternoon nap, but sometimes when you feel tired you need the nap. She said no you have got enough sleep you don't need the afternoon nap. So basically she was just teaching me how to sleep again. It has been a long time since I knew how to sleep… I don't know why, maybe it is [the therapist] that helped me, maybe because of her and I helped myself. I tried what she suggested and it did work."

Patient 5

"Yes, she gave me quite a lot of tips actually. She gave me a lot of tips that is what we did most of the time. Tips on turning off the TV. We talked about not sleeping and relaxing in the same place, so I could tell the difference between time sleeping and time relaxing…I am doing a lot more important stuff in the week than I was before and I do a whole lot more important things."

Sensitivity analysis (adjusting for baseline score, PANSS score, and baseline medication) for the primary outcome measures

		CBT group		Control group		
	**Week**	**n**	**Adjusted mean (95% CI)**	**n**	**Adjusted mean (95% CI)**	**Adjusted mean difference (95% CI)**
**Insomnia Severity Index**	12	22	9.3 (6.9, 11.6)	25	15.5 (13.3, 17.6)	6.2 (3.0, 9.4)
	24	23	11.0 (8.9,13.2)	25	15.1 (13.0, 17.1)	4.0 (1.0, 7.1)
**PSYRATS Delusion**	12	22	13.6 (12.0, 15.3)	25	14.1 (12.5, 15.6)	0.5 (-1.8,2.7)
	24	23	13.7 (11.6,15.7)	25	13.0 (11.0, 15.0)	-0.7 (-3.5, 2.2)
**PSYRATS Hallucination**	12	22	27.5 (24.4, 30.7)	25	25.9 (23.0, 28.9)	-1.6 (-6.0, 2.8)
	24	23	25.2 (21.0, 29.5)	25	21.4 (17.3, 25.5)	-3.8 (-9.8, 2.2)

Correlation coefficients between changes in insomnia and change in psychotic experiences at 12 weeks

Psychosis difference score	Statistics	ISI difference score		
		**Both groups**	**CBT**	**Control**
**PSYRATS Delusion**	Rho	.01	.04	-.12
	p-value	.959	.845	.576
	n	47	22	25
	95%CI	-.28, .29	-.38, .46	-.49, .29
**PSYRATS Hallucination**	Rho	.26	.35	.41
	p-value	.078	.111	.041
	n	47	22	25
	95%CI	-.03, .51	-.09, .67	.02, .69
**PTS Paranoia**	Rho	.16	.08	.22
	p-value	.280	.717	.309
	n	46	22	24
	95%CI	-.13, .43	-.35, .49	-.20, .57

Provision of additional psychiatric medication

	CBT group		TAU group	
	**Prescribed (n)**	**Not prescribed (n)**	**Prescribed (n)**	**Not prescribed (n)**
**Hypnotics**				
Baseline	6	18	3	23
12 weeks	6	17	4	21
24 weeks	6	17	5	20
**Anxiolytics**				
Baseline	5	19	9	17
12 weeks	5	18	7	18
24 weeks	3	20	9	16
**SSRI anti-depressant**				
Baseline	15	9	14	12
12 weeks	13	10	13	12
24 weeks	13	10	13	12
**Tricyclic anti-**				
**depressant**	1	23	2	24
Baseline	1	22	2	23
12 weeks	0	23	2	23
24 weeks				

Mixed models for primary outcome measures, using an unstructured covariance structure

In a post hoc analysis, we constructed a mixed model to incorporate the repeated measures at the two assessments (weeks 12 and 24) for each patient, using PROC MIXED in SAS. Treatment (CBT v.s. TAU), assessment, treatment-and-assessment interaction, and baseline effects were fitted as fixed effects. The restricted maximum likelihood (REML) method was used to estimate the model parameters, and an unstructured covariance structure was used to model the within-patient errors. The Kenward and Roger method was used to compute denominator degrees of freedom and adjust the downward bias of fixed effect standard errors. The results using the mixed model are very similar to the results obtained using the ANCOVA shown in the main paper (table 2).

		CBT		TAU		
**Variable Name**	**Week**	**N**	**Mean (95% CI)**	**N**	**Mean (95% CI)**	**Mean Difference (95% CI)**
**ISI**	12	22	9.4 (6.64, 12.08)	25	15.4 (13.55, 17.23)	6.0 (2.83, 9.23)
	24	23	11.1 (8.79, 13.43)	25	15.0 (13.04, 16.94)	3.9 (0.93, 6.83)
**PSYRATS Delusion**	12	22	13.5 (11.59, 15.41)	25	14.0 (12.53, 15.51)	0.5 (-1.84, 2.88)
	24	23	13.7 (11.76, 15.64)	25	12.9 (10.80, 15.00)	-0.8 (-3.59, 1.99)
**PSYRATS Hallucination**	12	22	27.3 (22.94, 31.58)	25	25.6 (23.15, 28.05)	-1.7 (-6.52, 3.20)
	24	23	25.3 (20.34, 30.18)	25	21.7 (18.33, 25.03)	-3.6 (-9.39, 2.23)
